# The UK cost-of-living crisis and its effect on students at a British medical school

**DOI:** 10.1186/s12909-025-07305-5

**Published:** 2025-05-28

**Authors:** Shanzeh Nadim Iqbal, Amanjeet Rekhi, Arun James, Jo-Anne Johnson, Aaron Trinidade

**Affiliations:** 1 Anglia Ruskin University School of Medicine, Chelmsford, UK; 2 Department of Otolaryngology, Mid & South Essex NHS Trust, Southend Hospital, Southend-On-Sea, UK

**Keywords:** Cost-of-living- crisis, Medical students, Medical education, Financial stress

## Abstract

**Background:**

The current cost-of-living crisis in the UK has negatively affected wide sections of society, including university students.

**Aim:**

To determine how the lifestyle, behaviors, mental health and education of students at an English medical school have been affected by the crisis.

**Methods:**

Mixed methods cross-sectional study.

**Results:**

One hundred and forty-seven students responded to the questionnaire; 53% (*n* = 106) receiving maintenance loans reported that their loans only covering up to half of their costs; 72% (*n* = 103) felt the need to either gain employment or increase their current working hours; 75% (*n* = 110) reported gaining income through employment, 55% (*n* = 57) of which agreed or strongly agreed with their work having a negative impact on their studies; 83% (*n* = 87) reported significant increases in the amount spent on rent, household bills, and food; 69% (*n* = 73) restricted their spending on essentials such as food, heating, and clothing.

**Conclusion:**

There is an urgent need for increased financial support for medical students in the current cost-of-living crisis, particularly those from lower-income families, first-generation students, and those entering through widening access programmes. The current financial aid structure, combined with the pressures of the cost-of-living crisis, is insufficient to meet students' basic needs and is compromising their education, well-being, and professional development.

**Supplementary Information:**

The online version contains supplementary material available at 10.1186/s12909-025-07305-5.

## Introduction

As of April 2023, 93% of adults in Great Britain reported a rise in their cost of living, according to the Office for National Statistics. The Office for Budget Responsibility projected a 4.3% decline in real post-tax household income for 2022–23, marking the largest drop since records began in 1956. Food prices also surged, rising 19.1% in March 2023 compared to the previous year, a 45-year high [1, 2]. This surge has resulted in widespread financial difficulties, including among medical students.

In the context of these challenges, a UK-wide student survey conducted by the British Medical Association (BMA) in April and May 2022 revealed that 44.3% of the 1,119 students surveyed expected to run out of money before the academic year ended. Furthermore, 61.8% reported cutting back on essentials such as food, heating, and clothing, while nearly 1 in 25 students reported accessing food banks. For those eligible for NHS bursaries, the support covered only 30% of their expenses. Additionally, more than half (53.6%) of students worked during term time, with 73.1% reporting that their work negatively impacted their studies; 28.5% received no reimbursement for placement travel expenses. This financial strain affected students across England, Wales, and Scotland equally [3].

The aim of this mixed-methods cross-sectional study was to assess the effect that the cost-of-living crisis had on the quality of life of students at a UK medical school in 2024. The study explores the experiences of medical students as they navigate financial pressures, and how these challenges impact their academic and personal well-being. The study builds upon the themes explored in the 2022 BMA Student Survey, examining whether these trends have persisted while delving into greater depth than previous research.

### Methodology

Medical students from Years 1 to 5 at Anglia Ruskin University (ARU), located in Essex, England, were invited to complete an online survey via Microsoft Forms in January 2024. At the time, the university had 528 enrolled students. Students from all 5 years were invited to participate in the survey which was delivered online via the Jisc platform (www.jisk.ac.uk). The survey was constructed based on the collective experiences of Year 4 medical students (including authors SNI, AR and AJ) and through informal discussions with student peers, ensuring relevance of the addressed issues. Quantitative and qualitative questions were employed to capture a broad spectrum of personal and academic challenges posed by the cost-of-living crisis. This allowed the quantification of specific hardships but also exploration of the nuanced ways in which the crisis has affected daily living and academic performance amongst student peers. Data collection lasted for 20 weeks. All students were eligible to participate, with no exclusion criteria. The survey consisted of five sections: demographics, sources of income, cost of living, work and education-related expenses, and financial stress/quality of life. Financial stress and quality of life were investigated through survey questions as opposed to using validated tools.

Demographic data collection included students'age, sex, year group, Widening Access to Medical School (WAMS) classification, and socio-economic status, using the National Readership Survey (NRS) social grade. WAMS is an initiative at ARU that is part of a national effort to encourage and support students from underrepresented or disadvantaged backgrounds to pursue a career in medicine. The survey included multiple-choice, closed, and open-ended questions, allowing students to share their experiences related to the cost-of-living crisis (Appendix 1). The open-ended questions provided the qualitative aspect of the study.

### Statistical analysis

Data were analysed using SPSSv29 and Excel version 16.50. Quantitative data analysis was used to interpret the closed question responses, and a thematic analysis was performed on the qualitative free-text responses in each of the categories excluding the demographics section. Continuous non-parametric data were analysed for statistical significance across demographic groups using the Mann–Whitney U Test.

### Qualitative analysis

With respect to the qualitative data, reflexive thematic analysis was employed: no predefined coding frame was used but was instead iterative and dynamic and themes were developed from an interpretive process. The data was read and re-read to achieve immersion and obtain a sense of the whole. Codes were then generated by marking ideas directly to the data, then collating themes by grouping related coded data extracts. This was an iterative process, as themes were continuously refined to ensure they reflected the coded extracts and the entire data set. To ensure rigor and reduce bias, three researchers independently coded the data, discrepancies were discussed and a consensus reached, thus mitigating personal biases and enhancing reliability. Additionally, to enhance the validity of the findings, direct quotes were used as illustrative examples of the themes, providing a transparent link between data interpretation and the data itself.

## Results

### *Demographics (*Table 1*)*

**Table 1:** Demographic data of students completing the survey

		**n**	**%**
**Age**	18–20	46	31.3%
	21–24	80	54.4%
	25–28	14	9.5%
	29–34	5	3.4%
	25–38	1	0.7%
	39 +	1	0.7%
**Sex**	Male	103	70.1%
	Female	43	29.3%
	Prefer not to say	1	0.7%
**Year group**	Year 1	36	24.5%
	Year 2	35	23.8%
	Year 3	26	17.7%
	Intercalating	2	1.4%
	Year 4	42	28.6%
	Year 5	6	4.1%
**Degrees prior to starting medicine**	Yes No	30 117	20.4% 79.6%
**Widening access to medicine (WAMS)**	Yes No	41 106	27.9% 72.1%
**Highest earning parent’s job**	ABC1 C2DE	122 25	83.0% 17.0%

A total of 147 (28%) students completed the survey. Most respondents were aged 21–24 (*n* = 80; 54%), male (*n *= 103; 70%). Medicine was the first degree for most (*n *= 117; 80%). Two NRS social grades were identified in our study: C2DE (lower-income or working-class; *n* = 25; 17%) and ABC1 (middle class to upper middle class; *n* = 122; 83%). The majority were not classified as WAMS (*n* = 106; 72%). First-generation students (*n* = 40; 27%) were defined as students who were the first in their family to attend university, as per the UK’s Universities and Colleges Admissions Service (UCAS) [4].

With respect to the qualitative results, 44 students highlighted other ways the cost-of-living crisis had affected them. Five major themes were identified: (1) mental and emotional stress; (2) family and personal relationships; (3) social life and extracurricular activities; (4) financial constraints and adjustments; (5) academic and professional decisions (Table 2). Where appropriate, both quantitative and qualitative results are presented in an integrated fashion under common themes to provide a comprehensive understanding of complex issues.

**Table 2:** Qualitative analysis of responses to open-ended questions

	**Codes**	**n**	**Extracts**
*Changes to living circumstances due to the cost-of-living crisis in the past 2 years*			
**Increased Housing costs and affordability**	Rent increase House affordability Student accommodation unaffordable Moved back home Living with family or friends Switching accommodations Room sharing	14	*“The cost of rent and living costs (food, transport, basic necessities) has hugely increased with no financial compensation to accommodate for these changes.”* *“More money spent on food; price of rent has increased.”* *“I had to move back home from private student accommodation on campus in order to save money on rent.”* *“Couldn’t afford to cover the university accommodation (quite pricey) and final year we have more expenditure (e.g. electives, full-time placement). We also have less student finance.”* *“At the end of this academic year, I will have to move back home and commute as rent has gone up.”*
**Rising utilities and living expenses**	Increased utility bills Increased food costs Transportation cost rise Budgeting for essentials Energy conservation	8	*“Aldi branded items, never turning the heat on, not turning the lights on, living in a mouldy place because it’s cheap, no fruits/veg because it’s expensive, don’t drive as too expensive, use the same laptop that doesn’t work as too poor to buy a new one.”* *“Increased living expenses such as electricity and gas bills.”* *“Don't turn on the heating.”*
**Work and financial adjustments**	Work more hour Debt accumulation Increased reliance on loans Struggle with tuition fees	4	*“I’ve had to work more to achieve the same living conditions regarding car insurance, food, *etc*.”* *“Energy bill from £60 to £220 a month. Mortgage from 800 to 1200. Fuel. Food. I have zero spare money and survive on credit cards. The ‘bursary’ of £200 a year is literally less than 2 weeks food.”* *“We are struggling to pay for rent. I am also a new mother and whatever money I receive from maintenance goes towards nappies, food, baby things. I have very little left to cover my tuition fees. I must pay tuition fees as a grad with NO bursary support. This university gave £100 for bursary… that all went on my transport to this campus… in 2 days. A complete joke. There is no financial support or accessibility. Even the head of medical school was not receptive to our complaints.”*
**Financial strain on students**	Student financial strain Lack of financial support Struggle with tuition fees	5	*“Energy bill from £60 to £220 a month. Mortgage from 800 to 1200. Fuel. Food. I have zero spare money and survive on credit cards. The ‘bursary’ of £200 a year is literally less than 2 weeks food.”*
**Reduced quality of life and mental health impact**	Reduced quality of life Social life, low mood, anxiety Limited social activities	3	*“I have had to be hyper aware of putting the heating on, using electricity less freely.”* *“I can’t afford the small joys in life such as going to a coffee shop, eating out with friends. This has really affected my mental health especially as the course is tough and I need an outlet for it.”*
*Changes in expenditure due to the cost-of-living crisis*			
**Changes in spending habits**	Shopping in bulk or at cheaper stores Reduced spending on non-essentials (e.g., clothes, eating out) Prioritizing essentials over leisure activities Cutting back on food quality or quantity Switching to cheaper alternatives	54	*“I have been eating unhealthier as I can’t afford food produce.“* *“Eating more vegetables and not meat.”* *“Avoiding turning on heating in winter and just buying a blanket.“* *“Eating only 1 kg bag of dry pasta toward end of term.“* *“Avoiding buying placement clothes and just using scrubs.”* *“More frugal with heating, food, activities.”* *“Shopping in charity shops, not going out socially. Not eating out bar birthdays. Buying frozen food always. Having left over food from work as dinner.“*
**Impact on daily life**	Reduced social activities Limiting travel due to high costs Increased planning and budgeting for necessities Sacrificing essentials like heating and food	40	*“No longer able to save for the future … spend more time planning (and worrying) about shopping for food.”* *“I have been eating unhealthier as I can’t afford food produce … Fewer takeaways and less clothes shopping.”* *“Trying to spend less on food shops, limiting turning on heating.”*
**Accommodation and travel**	Moving back home or finding cheaper accommodation Spending more time in free spaces like libraries to save costs Reducing or avoiding travel due to high costs Sacrificing comfort to afford rent or bills (e.g. no heating) Spending more time in free spaces like libraries to save costs	31	*"Rent has taken a much bigger increase … limited food shopping budget — hence living on pasta and non-perishables!"* *"Having to spend more time in the library as it’s too expensive to turn on the heater in our house."* *"By the end of the semester, I'm usually running very low on money … petrol costs mean I am spending more on average."* *"I can’t go out as much or do things with friends or even visit my family due to travel costs."*
**Financial strain and employment**	Increased financial burden due to static or insufficient income Inability to save money Relying on family for financial support Considering dropping out or taking on additional work	18	*"All my income from student finance and from tutoring goes towards essential bills, rent, and food … I can’t afford to spend money on anything else."* *“Seeking financial support from family to cover basics; travel.”* *“Seriously considering going back to work and dropping out of studying.”*
**Impact on physical and mental wellbeing**	Increased stress and worry about finances Negative impact on physical health due to poor diet Reduced mental well-being due to financial strain Feeling isolated or limited in social interactions	11	*"I found my energy levels and general mood were both lowered due to the fact that I was eating worse and also … had to think about what I could get away with buying."* *“I often find myself limiting my own activities because I cannot afford to pay to go out.”* *“By the end of the semester, I'm usually running very low on money … I often find myself picking between items … and weighing up whether or not I could afford to pay for any extra if I wanted to make rent."* *"I now only spend on essentials … can’t afford food on campus so long days of lectures are difficult."* *"I have been eating unhealthier as I can’t afford food produce."*
*Financial challenges faced at placement*			
**High travel and transport, and material costs**	Expensive Travel Inadequate Reimbursement Limited Transport Options Inadequate reimbursement Limited transport options	27	*“Traveling to placement and material to aid me in study for some of my rotations, where purchase of question banks or knowledge banks are essential.”* *“It is more money to run your car than it was 3 years ago but we have been paid the same travel bursary costs as what the now graduated cohort received all those years ago.”* *“To get an Uber would be 40 quid one way, which I can’t really spend in a day and therefore have missed GP placement a couple of times.”* *“To attend GP placement for the three days that I attended, it cost around £60.”* *Could barely pay for travel and upon arriving to GP placement they turned me away because they “weren’t expecting me.”*
**Delayed and insufficient travel bursaries**	Delayed Bursary Payments Self-funding Increased costs	14	*“The travel bursary arrived midway through first rotation of the year.”* *“…I was scared to take train cos it’s a lot of money…”* *“Bursary placements are slow so “money that was supposed to be used for other things was used for placement…. Had to work more in the meantime and really struggled with balancing placement, work, uni, SSC and being able to have a life of my own.”* *“Had to borrow money to pay for travel to placement.”* *“Work more hours and making lifestyle sacrifices.”* *“Having to pay for travel out of my maintenance loan put me behind on my rent.”*
**Financial strain and borrowing**	Borrowing Money Additional Work Hours	35	*“Received funding late, so had to fund myself.”* *“Having to pay for travel out of my maintenance loan put me behind on my rent.”* *“Delayed deposits which lead to anxiety and overhauls.”* *“I have regularly been in and out of my overdraft during my studies.”* *“Having to ask family to pay for it as wouldn’t be able to buy food.”*
**Impact on academic and personal life**	Balancing Work and Placement	13	*"Delay in travel bursary led to having to work more hours and making lifestyle sacrifices to afford to travel to placement."* *“On our 5-week placement blocks I was forced to work 12 h shifts on the Saturday and Sundays. Every week.”*
**General cost of living challenges**	Lifestyle sacrifices	8	*‘Abrupt housing changes. Having a dependent. Less opportunities to work as well.”* *“Placement clothes are so expensive, as is washing them regularly to maintain infection control.”* *“Placement clothing can be expensive and isn’t always possible to get scrubs that fit."*
*Effect of the cost-of-living crisis on studies*			
**Increased financial stress and anxiety**	Worry about money Constant financial pressure Struggle to pay rent and bills Financial distractions Stress from basic expenses	13	*“More worried about money.”* *"It’s been a constant stress in the back of my mind, and I can’t focus on my studies."*
**Balancing work and studies**	Working to cover costs Less study time due to work Balancing job and studies Work affecting academic focus Skipping lectures for work	25	*"Overall, it’s affected my mental health more than anything which has had a significant impact on my studies and motivation to continue the degree."*
**Impact on mental health**	Increased anxiety Worsened mental health Strain affecting anxiety Seeking mental health support Mental health hurting academic motivation	11	*"Just impacted my mental health and causes financial stress."* *“Considerably more stressed and anxious, have sleepless nights worrying about money.”*
**Reduced study time and academic performance**	Study time cut by work Less time for revision Exhausted from balancing work and study Missing study to earn money Struggling to find time to study	21	*”Less time to study as I need to work.”* *“Reduction of ability to spend time on academics due to increased working hours.”* *“Having to work instead of going over the lectures and the workload ends up piling up, causing more stress and anxiety.”* *“I am having to work so many hours to fund living and tuition that I’m often too exhausted to study and engage with content in the way I would like.”* *“I work so much, especially since the cost-of-living crisis. I don’t have time to study apart from late at night.”*
**Inability to afford educational resources and extracurricular activities**	Inability to afford study materials Unable to attend conferences Financially limited access to resources Skipping extracurriculars due to cost	15	*“Sometimes can't go to conferences due to financial reasons and starting to feel left behind due to financial costs."* *“Not being to able to afford study materials (certain books, website subscriptions for revision, paid apps:”* *“I can’t always afford equipment, books and access to revision sites to aid revision that others have access to. Unfortunately, some of the best revision resources are paid for.”* *“Hard to pay for resources for study, general financial stress regarding food and rent, having to work takes time from studying.”* *“I can’t even afford to commute or the food here. I literally have no financial support.”*
**Changes in living arrangement**	Moved home due to cost Cant afford student housing Living situation impacts study Increased housing and travel costs	4	*“I have had to move home and choose an elective in the UK, in addition to working part time in term time during year 2 and 3 which impacted the number of hours I had available to study."* *"I wanted to move out to student accommodation for the academic year, but I was unable to due to the costs, and I feel that I was able to study better when I was living on campus, compared to now living at home due to the commute."* *“I have failed exams as unable to take time off. Feel I could become homeless if I don’t."* *"I wanted to move out to student accommodation for the academic year, but I was unable to due to the costs, and I feel that I was able to study better when I was living on campus, compared to now living at home due to the commute."* *"During my repeated year of study, I had to work more to afford accommodation."* *"I have a family to support and the increased costs of living, increased mortgage rates and travel prices have meant studying is more costly than initially expected. I struggle to ensure my family are comfortable."*
*Other ways cost of living crisis has affected students*			
**Mental and emotional stress**	Stress Burnout worry Mental health toll isolation	15	*“Mentally I feel so stressed, and I often have depressive thoughts because I feel as if I can’t cope anymore. Studying a course this intense combined with the current cost of living and trying to balance being able to pay bills with employment or risk affecting my studies has been extremely difficult and taken a huge toll on me mentally!”* *“Increased stress and reliance on parents for support, increased deb.t”* *“Just feel generally more worried about finances and if I'll have enough money to see me through. Also worried about funding my own transport in further years when we have placement only. Accommodation is also not cheap in Chelmsford and there are barely any options anyway so all that plays on my mind a lot.”*
**Financial constraints**	Spending cuts Budgeting savings debts Rising costs	13	*“Have just had to be more practical when making decisions (e.g. no intercalation due to cost).”* *“Spending less on food and clothes.”* *“Think a lot more about spending.”* *“Unable to save any money.”*
**Family and personal relationships**	Reliance on family Distance from home Emotional burden, guilt due to reliance on family Missed holidays	10	*“Meant I have spent Christmas and summer holidays working in Chelmsford away from family.”* *“Can’t go home as much.”* *“Feel like a burden to my family.”* *“Unable to travel to see my family, less able to do extracurricular activities including gym membership due to prices and timings of the university gym not being flexible for my work-uni schedule *etc*.”*
**Academica and professional decisions**	Intercalation decisions Work-study balance Funding concerns Career impacts/sacrificing academic opportunities	10	*“Decided not to intercalate as I need to start a job.“* *“No work life balance.”* *“I am very concerned about how I will fund my year 5.”*
**Social life and extracurricular activities**	Less socialising Reduced extracurriculars Isolation Missed opportunities	8	*“Reduced social life due to cost.”* *“It is a very intense course, so it is important to take days off and time out, it is much harder to do this when there is very little money to spend.”* *“Unable to enjoy my university experience as I don’t have the money to spend.“*

### Sources of income

**Fig. 1:**
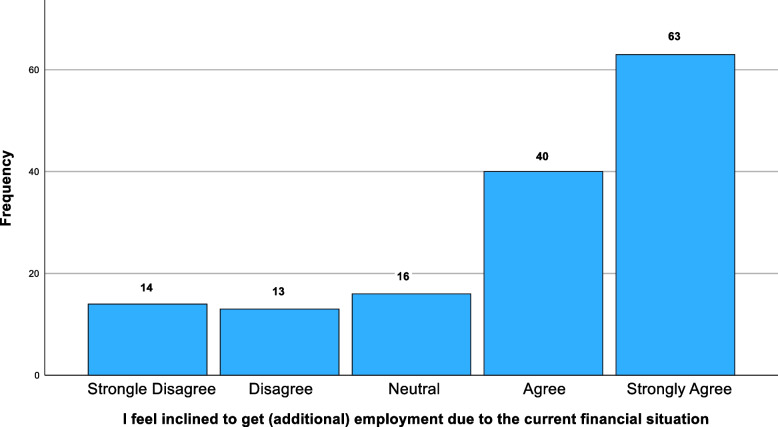
Percentages of students who felt inclined to get additional employment tdue to their current financial situation

72% (*n* = 106) of participants, including 19 graduate students, received loans, of who 47% (*n* = 49) found that it covered less than half of living expenses (Fig. 1). Most working-class students 76% (*n* = 19) received loans, with 53% (*n* = 10) having loans that covered at least 60% of their expenses, compared to 37% (*n* = 32) of middle/upper class students, with 71% (*n* = 87) received loans providing equivalent expense coverage (*p* = 0.967).

75% of students (*n* = 110) gained additional income through employment (Fig. 1), which 55% (*n* = 59) felt adversely impacted their studies. Employment was higher in first-generation (83%, *n* = 23) and WAMS (78%, *n* = 32) students. Loans also had slightly better coverage in first-generation students, with 51% (*n* = 12) having more than 60% covered compared to non-first-generation students (*p* = 0.925). Similarly, WAMS students also received better loan coverage compared with non-WAMS students, with 47% (*n* = 32) having over 71% of costs covered (*p* = 0.003).

### Impact of increased cost of living on personal circumstances and wellbeing

At the time of the study inflation was at 5.1%. Since 2022, 27% (*n* = 40) of participants reported changing their living circumstances: 72% (*n* = 106) live either in student accommodation or private rentals, with 67% (*n* = 96) reporting spending over 70% of income on rent, household bills, and food, which have seen substantial increases in expenditure for 83% (*n* = 121) of students compared with the previous year. Several students have families of their own, with one stating, “We are struggling to pay for rent. I am also a new mother and whatever money I receive from maintenance goes towards nappies, food, baby things. I have very little left to cover my tuition fees. I must pay tuition fees as a grad with NO bursary support. This university gave £100 for bursary… that all went on my transport to this campus… in 2 days. A complete joke. There is no financial support or accessibility.” And another,"I have a family to support and the increased costs of living, increased mortgage rates and travel prices have meant studying is more costly than initially expected. I struggle to ensure my family are comfortable."

Thirty-seven students struggling with higher rents sought cheaper alternatives, including moving back home. One student stated that the financial strain made them, “[I] feel like a burden to my family.” Eight cited rising utilities, food and travel expenses leading to bulk buying, reducing the number of daily meals and maximising time in the library or public spaces to reduce energy expenditure. There was an overall sense that students felt they were living in a state of relative poverty: “Shopping in charity shops, not going out socially. Not eating out bar birthdays. Buying frozen food always. Having left over food from work as dinner.” Over a third (36%) cited extreme frugality with heating and shifting toward eating less or unhealthier food to reduce expenses.

First-generation students were significantly more likely to report changes in their living circumstances (*p* = 0.012). Similarly, WAMS students experienced higher essential expenditures, with half (*n* = 20) spending over 90% of their income (*p* = 0.012). Social class did not appear to influence spending patterns, as middle/upper class and working-class students allocated a similar proportion of their income to expenses (*p* = 0.204). However, working-class students were notably more likely to report changes in their living circumstances, with 64% affected compared to just 20% of middle/upper class students.

### Impact of increased cost of living on education and professional development

**Fig. 2:**
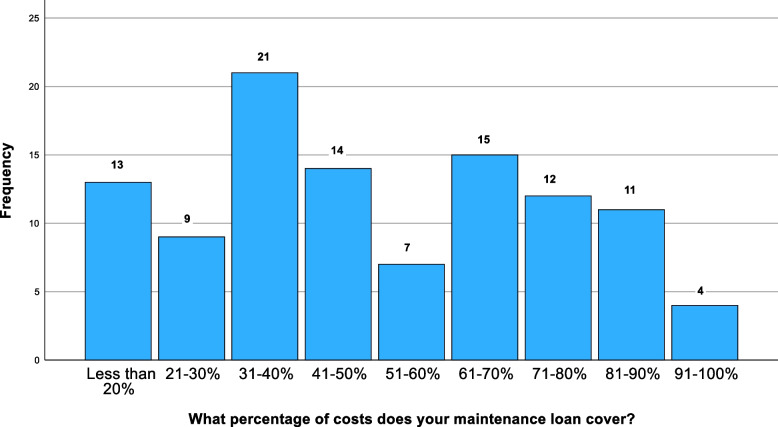
Percentage of costs that student maintenance loans covered

**Fig. 3:**
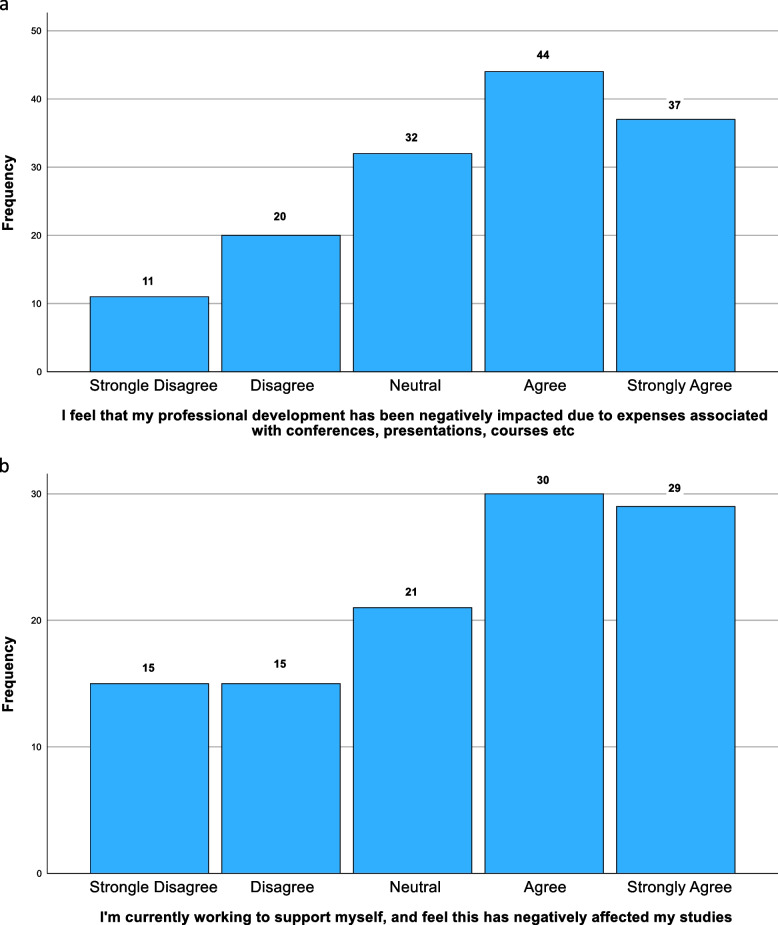
Bar charts showing (a) the negative impact that increased expenses has had on educational activities, and (b) the negative effect that additional employment has had on educational activities

Half of the students (*n* = 73) felt that their studies had been affected; 66% (*n* = 96) reported difficulty affording essential study materials (Fig. 2). Travelling to placement had become more expensive, with 47% (*n* = 64) struggling to afford it (Fig. 3). Only 26% (*n* = 36) reported fully funded travel, with 34% (*n* = 46) reporting having to self-fund travel entirely. Highlighted issues included delayed travel bursaries (23%), high travel and transport costs (44%), financial strain (57%), and the general high cost of living (13%). 43% (*n* = 62) reported that finances impacted their decision to intercalate, with 20% (*n* = 30) reporting a significant influence. Typical quotes include: “Sometimes [I] can't go to conferences due to financial reasons and [am] starting to feel left behind due to financial costs,"and, “[I cannot] afford study materials (certain books, website subscriptions for revision, paid apps),” and, “I can’t always afford equipment, books and access to revision sites to aid revision that others have access to. Unfortunately, some of the best revision resources are paid for.” 18% (*n* = 26) considering dropping out of the course altogether: “Seriously considering going back to work and dropping out of studying.”

**Fig. 4:**
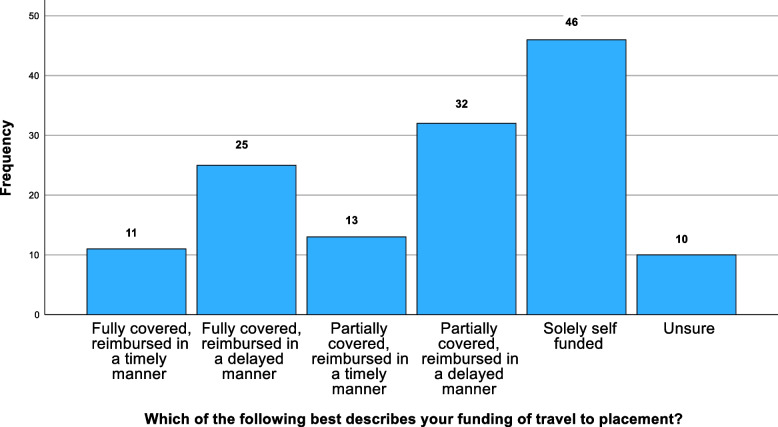
Funding sources for travel to placement

Most students reported negative impacts on professional development. WAMS students were statistically more likely to report negative impacts on professional development (*p* = 0.047). The role of social class did not receive statistical significance (*p* = 0.124) Fig. 4.

### The impact of cost-of-living crisis on financial stress and quality of life

**Fig. 5:**
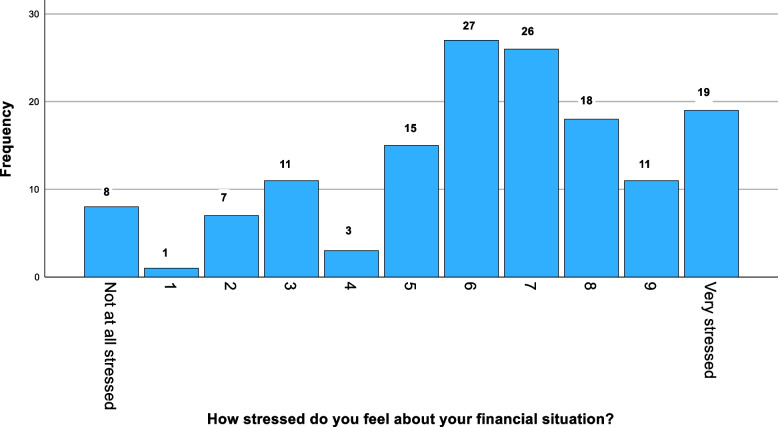
Stress levels in students with respect to the cost-of-living crisis

Participants rated their financial stress at a mean average of 6.3 out of 10. Overall, only 5% (*n* = 8) students out of 146 reported experiencing no levels of stress regarding their financial situation (Fig. 5). There was no significant difference in financial stress between first-generation and non-first-generation students (*p* = 0.418). However, WAMS students, who reported a mean financial stress of 8.7 compared to 6.8 for non-WAMS students, were more likely to report a poorer quality of life (*p* = 0.016), as were working-class students, with a mean score of 9.4, compared to middle/upper class students, with a mean score of 7.0 (*p* = 0.010)."

Five students reported a lack of support causing financial strain, increased stress, reduced leisure time, and burnout; 18% (*n* = 8) reported limiting their own activities as they could not afford to go out. One student stated, “It is a very intense course, so it is important to take days off and time out, it is much harder to do this when there is very little money to spend.” Another said, “Unable to enjoy my university experience as I don’t have the money to spend.” Even simple pleasures were seen as unaffordable luxuries: “I can’t afford the small joys in life such as going to a coffee shop, eating out with friends. This has really affected my mental health especially as the course is tough and I need an outlet for it.”

34% (*n* = 15) of responses highlighted mental and emotional stress, with it being the most prevalent theme, highlighting stress, anxiety, and worsening mental health due to finances, some reporting burnout; 20% (*n* = 13) reported worsened mental health; 17% (*n* = 11) reported anxiety, depression, and the need for professional support with their mental health. Quotes include, “Mentally I feel so stressed, and I often have depressive thoughts because I feel as if I can’t cope anymore. Studying a course this intense combined with the current cost of living and trying to balance being able to pay bills with employment or risk affecting my studies has been extremely difficult and taken a huge toll on me mentally!” and, “Just feel generally more worried about finances and if I'll have enough money to see me through. Also worried about funding my own transport in further years when we have placement only. Accommodation is also not cheap in Chelmsford and there are barely any options anyway so all that plays on my mind a lot.”

Support from family and social networks also suffered, further aggravating mental health. “… I have spent Christmas and summer holidays working in Chelmsford away from family,” “Can’t go home as much,” “Unable to travel to see my family, less able to do extracurricular activities including gym membership due to prices and timings of the university gym not being flexible for my work-uni schedule etc.,” were typical quotes. Many students felt they were neglecting their families due to reducing visits home as they would struggle to, “… balance placement, work, university….”

## Discussion

This study explored financial challenges faced by medical students at a single British medical school during a cost-of-living crisis, particularly in relation to income sources, ability to meet the cost of living, resulting financial stress, and the impact on their personal circumstances and wellbeing, education and professional development, as well as quality of life.

In terms of demographics, ARU School of Medicine demonstrates a high proportion of students from lower socio-economic backgrounds. In this study, a substantial proportion of students (72%) reported receiving student loans, with three-quarters of all responding medical students during the cost-of-living crisis supplementing their loans through employment to cover living expenses. This financial strain has broad-ranging implications, impacting personal circumstances and wellbeing, education, financial stress, and quality of life.

The findings of this study align with broader themes identified in the 2022 BMA Student Survey, which highlighted challenges such as inadequate student loan coverage, financial barriers to covering travel costs, the adverse impact of extra employment on academic development, and difficulties affording essentials. While these themes are broadly repeated in this study, the BMA survey’s broader scope did not evaluate how these challenges vary across key demographic groups or manifest in individual student experiences. This study adds depth and context by examining these issues in detail, particularly their disproportionate effects on first-generation and WAMS students, confirming their persistence as significant problems.

One finding of our study is that most students observed an increase in the cost of living compared to the previous year. This aligned with national data in 2024, when 92% of UK households reported increased food costs, and 68% reported higher energy bills [1, 5]. In terms of personal circumstances and wellbeing, this rise in costs disproportionately affected first-generation and WAMS students. The financial pressures led to changes in living arrangements, reductions in leisure and extracurricular activities, skipping meals, and taking extreme measures to minimise heating expenses. These adjustments reflect the challenging financial decisions students are having to make during the COL crisis, and the impact on wellbeing.

From an educational and professional development perspective, the cost-of-living crisis has profoundly impacted students. Half of all respondents reported that their studies had been adversely affected, with challenges accessing educational materials and affording transportation to placements. Most students acknowledged that their professional development had been hindered, with financial pressures discouraging them from pursuing academic opportunities outside the core curriculum. Specifically, students were less likely to intercalate onto external degree courses during the cost-of-living crisis, opting instead for the more financially viable option of completing their medical degree without delay. In addition to rising costs affecting education, employment also had a significant impact on students’ studies. Over half of employed students reported that their work negatively affected their education, with WAMS students and first-generation students being particularly impacted.

This study revealed that quality of life was reported to be lower among WAMS students and those from lower socio-economic backgrounds. These students also reported higher financial stress scores, highlighting the connection between financial stress and quality of life. We propose that all the factors examined in this study—financial strain, employment, education, and personal circumstances—can negatively affect quality of life. Given that WAMS students experience more significant challenges in each of these areas compared to their non-WAMS peers, it is unsurprising that they report a lower overall quality of life.

The negative impact of financial difficulties on medical students’ mental health is well-documented in the literature. For instance, a 2020 study by Shao et al. [6] found that 57.5% of Chinese medical students experienced depression, with higher rates among those under financial stress. In our study, students reported that relying on parental support often led to feelings of guilt, with some even questioning their ability to continue their studies. Alarmingly, one-third of students reported mental health challenges linked to the cost-of-living crisis, with 17% requiring professional mental health support. These findings likely reflect the cumulative effects of financial strain, including working alongside studies and dependence on parental contributions, on the various aspects of quality of life explored in this article.

Lastly, further evaluation is needed to determine whether students are fully aware of the financial resources available to them through the university and external organisations. Increasing the accessibility of information about these resources and providing more financial support, such as subsidies for study materials and conference fees, could help alleviate some of the financial burdens faced by medical students [7].

### Limitations of the study

This study focuses on data from a single medical school, reflecting the experiences of a specific group of students at one institution. Consequently, the findings may not fully capture the experiences of medical students across the UK, particularly as the four nations of the UK have distinct approaches to university funding in general and medical student support specifically. Additionally, a comparison between responders and non-responders was not made, and so it is difficult to know whether the findings are representative of the broader student population at the school. However, given the alignment between this study’s findings and those of the broader BMA survey, it is reasonable to extrapolate these results to a wider context.

The demographic representation of male students in this study was 42%, compared to 47% at the university, suggesting that the sample was reasonably reflective of the overall male-to-female ratio at the institution. However, the overall response rate was 28% of the medical student population, which is relatively low and may introduce non-response bias. This low response rate could reduce the accuracy of the findings and limit the ability to generalise them to the entire student body. Moreover, students who did not participate, particularly those who may be facing greater financial distress, such as intercalating students with lower financial support through student finance and NHS bursaries, are underrepresented in this study. Their lack of participation may skew the results, as their unique financial challenges are not fully captured.

The cross-sectional design of the study, which captures data at a single point in time, further limits its ability to infer causality or observe how the cost-of-living crisis impacts students'financial stress and quality of life over an extended period. Longitudinal studies are needed to track these changes over time and provide a deeper understanding of how financial pressures evolve during a student’s medical education.

Another potential limitation is the data collection method, which relied on online university platforms and in-person promotion during lectures. This approach may introduce selection bias, as students who are more engaged with these communication methods may have been more likely to respond. Consequently, certain groups of students could be overrepresented, potentially skewing the findings. Additionally, while the survey addressed key aspects of financial income, expenses, and financial stress, it did not account for other potentially influential factors, such as personal savings, scholarships, or external financial support. These factors may play a critical role in shaping a student's financial experience and should be considered in future studies.

## Conclusion

This study highlights the significant financial challenges faced by medical students during the cost-of-living crisis, with particularly profound effects on WAMS students and those from lower socio-economic backgrounds. The findings demonstrate the far-reaching consequences of financial strain, spanning personal wellbeing and mental health, academic progress, professional development, and overall quality of life.

While previous research, such as the 2022 BMA Student Survey, has identified similar themes, this study offers a deeper examination of the unique vulnerabilities of specific demographic groups. It underscores the disproportionate impact on WAMS students, who reported heightened impact of employment on their education, personal circumstances, increased financial stress, and overall reduced quality of life.

The interplay between financial pressures and academic challenges is particularly concerning, as students often face difficult trade-offs, such as delaying professional development opportunities, limiting extracurricular engagement, or balancing employment with demanding academic commitments. These stressors not only hinder educational progress but also have significant implications for students’ mental health and long-term career aspirations.

Addressing these challenges requires systemic interventions to support students financially, reduce barriers to accessing essential resources, and promote mental health. Future research should further investigate the mental health implications of the cost-of-living crisis, particularly among WAMS students, and evaluate strategies to mitigate these effects to ensure equitable outcomes for all medical students.

## Supplementary Information

Supplementary Material 1.

## Data Availability

Data is provided within the manuscript or supplementary information files.

## Declarations

### Patient or public involvement

Patients or the public were not involved in the design, or conduct, or reporting, or dissemination plans of our research.
