# Supplementary material for: The UK cost-of-living crisis and its effect on students at a British medical school
Source: BMC Med Educ. 2025 May 28;25:791. doi: 10.1186/s12909-025-07305-5 (PMC12121211; doi:10.1186/s12909-025-07305-5)

CLIMB study

1. Consent to participate *

I agree to take part in the Cost-of-Living study and understand that my data will be processed according to UK GDPR guidelines. I also understand that my data cannot be withdrawn once submitted

2. Age *
*Drop down options*

- 18-20
- 21-24
- 25-28
- 29-34
- 35-38
- 39+
- Prefer not to say

3. Sex *

- Male
- Female
- Other
- Prefer not to say

4. Which year group are you in? *

- Year 1
- Year 2
- Year 3
- Intercalating
- Year 4
- Year 5

5. Do you have any previous degrees prior to starting Medicine?*

- Yes
- No

6. Are you the first in your family (parents, siblings etc) to attend university? *

- Yes
- No

7. Do you classify as a Widening Access to Medicine (WAMS) student? *
 *WAMS is a scheme aimed at helping those from under-represented/disadvantaged groups through the medical school selection process at ARU. Similar Widening Participation schemes may exist at other universities.*

- Yes
- No

8. Which of the following best describes your highest earning parents job? *

- Higher & intermediate managerial, administrative, professional occupation e.g. Chief executive, senior civil servant, doctor, bank manager, teacher etc.
- Supervisory, clerical & junior managerial, administrative, professional occupation e.g Shop floor supervisor, bank clerk, salesperson etc.
- Skilled manual occupation e.g Electrician, carpenter etc.
- Semi-skilled & unskilled manual occupation, or unemployed. e.g Assembly line worker, refuse collector, messenger etc.

Sources of Income

9. What is your main source of financial income?

- Maintenance loan
- Support from family
- Employment
- Bursaries

10. If you have any additional sources of income, please list them here


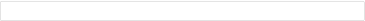


11. Do you receive maintenance loans, if so, what percentage of costs does it cover?

*Drop down options*

- I don’t receive student finance
- Covers less than 20%
- 20-30%
- 31-40%
- 41-50%
- 51-60%
- 61-70%
- 71-80%
- 81-90%
- 91-100%

12. Do you currently work part time/ full time? yes - full time during both term time and holidays

- yes - full-time during term time only
- yes - full time during term time and part time during holidays yes - part time during term time and full time during holidays yes - part time during term time only
- yes - full-time during holidays only
- yes - part time during term time and part time during holidays yes - part time during holidays only
- no

13. On average how many hours a week do you work?

*Drop down options*

- 1-5
- 6-10
- 11-15
- 16-20
- 21-25
- 26-30
- 31-35
- 26-40
- 40+

14. Has the Cost-of-Living meant that you have had to change your job or the amount of hours you work?


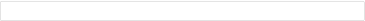


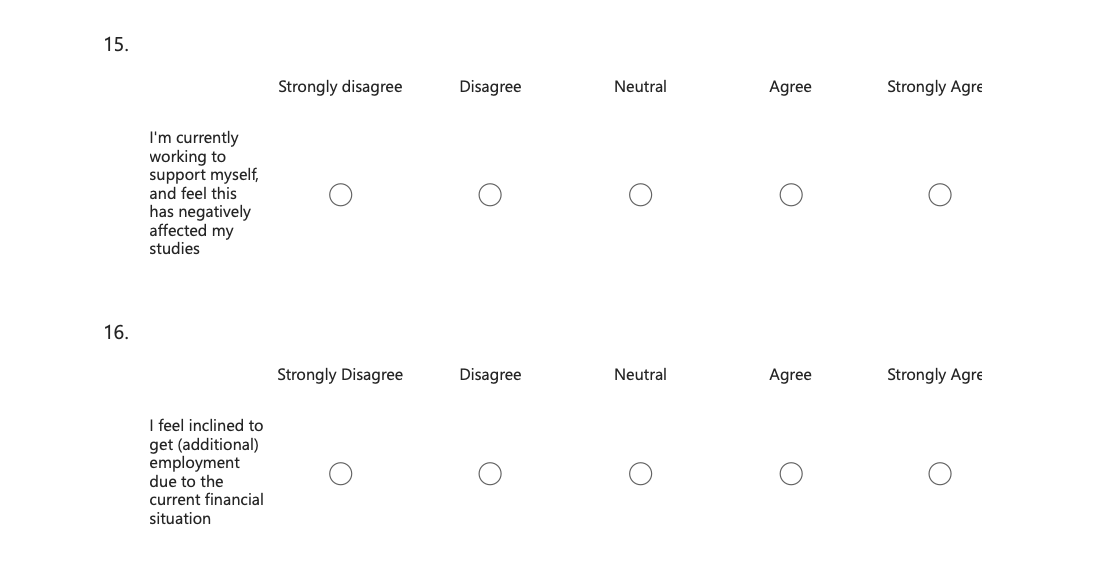


1. Do you think that you are eligible for financial support in the form of grants/ bursaries?

- Yes
- No

1. Do you access or receive bursaries from the university?

- Yes
- No

Cost of Living

1. Where do you currently live?

- Parental Home
- Student Accommodation
- Rent Privately
- Own property

1. Have your living circumstances changed due to the changes in the Cost-of-Living over the past 2 years?

- Yes
- No

1. If they have, please describe how your living circumstances have changed due to the Cost-of- Living in the past 2 years


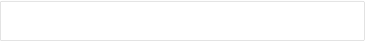


1. What proportion of income do you spend on rent, household bills and food?

0 1 2 3 4 5 6 7 8 9 10

<10% 100%

1. Has the proportion you spend on rent, household bills and food significantly increased in the last 2 years?

- Yes
- No

1. Have you restricted spending on essentials such as food, heating, and clothing in the last 2 years?

- Yes
- No

1. In what ways have you changed your expenditure due to the rising Cost of Living?


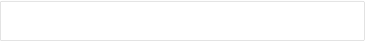


Work and Education Related Expenses

1. Do you feel less able to spend money on study materials, such as question banks and books, over the past 2 years due to the changes in the Cost-of-Living?

- Yes
- No

27. I feel that my professional development has been negatively impacted due to expenses associated with conferences, presentations, courses etc

- Strongly Disagree
- Disagree
- Neutral
- Agree
- Strongly Agree

1. Which of the following best describes your funding of travel to placement?

- My travel costs are fully covered, and reimbursed in a timely manner
- My travel costs are fully covered, but reimbursed in a slow or delayed manner
- My travel costs are only partially covered, and reimbursed in a timely manner
- My travel costs are only partially covered and generally reimbursed slowly or in a delayed manner Solely self-funded
- Unsure
- I have not had any placement this year

1. Have you faced any challenges funding your time at placement?

- Yes
- No

30. Please detail the financial challenges


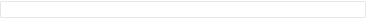


Financial Stress and Quality of Life

31. How stressed do you feel about your financial situation?

0 1 2 3 4 5 6 7 8 9 10

Not stressed Stressed

32. Has the Cost of Living in the recent years affected your studies?

- Yes
- No


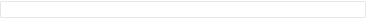
33. If so, please state how

34. Have you considered dropping out of university due to your financial situation?

0 1 2 3 4 5 6 7 8 9 10

No - never considered Yes- have thought about it many times

35. Has your decision to intercalate been impacted by your financial situation?

0 1 2 3 4 5 6 7 8 9 10

Not impacted Significantly impacted

36. In what other ways has the Cost of Living affected you?


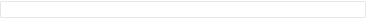

Supplement: Supplementary file 1 — Supplementary Material 1. [file 12909_2025_7305_MOESM1_ESM.docx]
